# Chronic Administration of Tadalafil Improves the Symptoms of Patients with Amicrobic MAGI: An Open Study

**DOI:** 10.1155/2017/3848545

**Published:** 2017-03-30

**Authors:** Sandro La Vignera, Rosita A. Condorelli, Laura M. Mongioi, Aldo E. Calogero

**Affiliations:** Department of Clinical and Experimental Medicine, University of Catania, Catania, Italy

## Abstract

Aim of this study was to evaluate the effects of pharmacological treatment with Tadalafil 5 mg daily on symptoms and quality of sperm parameters in selected patients with amicrobic MAGI (male accessory gland inflammation). 120 patients with amicrobic MAGI (mean age 27.0 ± 6.0 years) with mild-moderate ED (erectile dysfunction) according to IIEF-5 (International Index of Erectile Function 5 Items) scores underwent pharmacological treatment with Tadalafil 5 mg daily for six months. Before and after treatment these patients were evaluated through IIEF-5, semen analysis (according to WHO Criteria, 2010), SI-MAGI (Structured Interview about Male Accessory Gland Inflammation), and ultrasound evaluation. Patients with PVE (prostate-vesciculo-epididymitis) showed a significant increase in the percentage of spermatozoa with total (16.0 ± 8.0 versus 30.0 ± 6.0%) and progressive motility (8.00 ± 10.0 versus 25.0 ± 6.00%). It was a significant reduction of the number of patients with complicated ultrasound forms (30.0 versus 52.0) and a significant increase of the number of patients with uncomplicated ultrasound form (90.0 versus 68.0). Finally, there was a significant reduction in the percentage of patients with alterations of sexual function different from DE, such as premature ejaculation (4.00 versus 8.00%), painful ejaculation (4.00 versus 10.0%), delayed ejaculation (12.50 versus 8.00%), and decreased libido (10.0 versus 25.0%).

## 1. Introduction

The clinical presentation of patients with MAGI (male accessory gland inflammation) could be very heterogeneous; in fact, these patients may require andrological evaluation for problems (male infertility) [1, 2] and/or symptoms (chronic pelvic pain, ejaculatory and urinary disorders, and sexual dysfunction) [3]. Previously, we have shown that in the clinical practice, the use of a specific questionnaire [SI-MAGI (Structured Interview about Male Accessory Gland Inflammation)] helps to discriminate the differential diagnosis [4]. (Table 1 contains the questionnaire.)

Another very important aspect is the different anatomical extensions of the inflammation. From this point of view, MAGIs are classified into uncomplicated [P (prostatitis)] and complicated forms: PV (prostate-vesiculitis) and PVE (prostate-vesciculo-epididymitis). Generally, the complicated forms are associated with higher sperm abnormalities and increased severity of symptoms [4]. Moreover, the ultrasound evaluation represents an important tool for the differential diagnosis of complicated and uncomplicated forms [5]. ED (erectile dysfunction) and premature ejaculation represent the main sexual alterations in these patients [3]. This study assessed the changes observed after chronic daily treatment with Tadalafil (a specific inhibitor of V phosphodiesterase) regarding the symptoms and sperm parameters of the selected patients with MAGI.

## 2. Materials and Methods

One hundred twenty patients with amicrobic MAGI [1] were consecutively enrolled between 2013 and 2015, at the Institute of Andrology and Endocrinology of the University of Catania, Italy, aged between 20 and 45 years (mean age 27 ± 6.0 years) affected by mild–moderate ED, according to the scores obtained with IIEF-5 questionnaire (International Index of Erectile Function 5 Items) [6] administered during the first clinical evaluation.  Table 2 shows the diagnostic criteria of MAGI.

Patients were classified into uncomplicated and complicated forms, according to the ultrasound examination performed at enrollment (Table 1 contains the ultrasound criteria previously published) [5].

All patients were subjected to the following diagnostic procedure:

- Administration of SI-MAGI questionnaire [4]
- Sperm analysis after days of sexual abstinence [7]
- Ultrasound evaluation of scrotal and prostato-vesicular tract [5].

Diagnostic evaluation was performed at baseline and after six months of daily administration with Tadalafil 5 mg.

### 2.1. Exclusion Criteria

Endocrine and vascular alterations associated with ED (hypogonadism, hyperprolactinemia, obesity, diabetes mellitus, atherosclerosis, hypertension, and dyslipidemia). Patients with positive microbiological tests (semen culture, urine culture, HPV-DNA by PCR testing, and Stamey test).

The study was approved by the Internal Institutional Board, and all examined patients signed informed consent to the processing of personal data.

### 2.2. Statistical Analysis

Results are reported as mean ± SEM throughout the study. The data were analyzed by 1-way analysis of variance (ANOVA) followed by Tukey's test, as appropriate. The software SPSS 22.0 for Windows was used for statistical evaluation (SPSS Inc., Chicago, IL, USA). A statistically significant difference was accepted when the value was lower than 0.05.

## 3. Results

At baseline, according to the ultrasound criteria, sixty-eight patients had uncomplicated MAGI and the other fifty-two patients had complicated MAGI (thirty patients with PV and twenty-two patients with PVE). The mean value of the scores obtained with IIEF-5 was 14.0 ± 2.0. After therapy, a significant increase in the mean value of IEEF-5 score (23.0 ± 2.0 versus 14.0 ± 2.0) has been obtained.

After treatment, all domains of the SI-MAGI questionnaire were significantly improved in both groups (Table 3) and the percentage of spermatozoa with total and progressive motility was significantly improved in patients with PVE (Table 4). Finally, after treatment, according to the ultrasound criteria, ninety patients had uncomplicated MAGI (+32%) and another thirty patients (−42%) had complicated MAGI (twenty patients with PV and ten patients with PVE) (Table 5).

Other aspects of sexual function, different from erection quality, were significantly improved after pharmacological treatment (Table 5). In particular, the improvement is represented by the reduction of the percentage of patients who reported these symptoms evaluated through the SI-MAGI questionnaire (Table 6).

## 4. Discussion

The results of our study show that pharmacological treatment with Tadalafil 5 mg daily for six months, in selected patients with amicrobic MAGI and DE (not due to endocrine or vascular causes), is associated with significant improvements in sexual function, sperm parameters, and quality of symptoms reported by patients. In particular, the improvement of sexual function was assessed through the IIEF-5 questionnaire score that was significantly improved after treatment. Regarding the sperm parameters in patients with PVE (generally associated with lower quality of semen), there was a significant increase of sperm motility. Finally, with regard to other symptoms, the use of a specific questionnaire for these patients (SI-MAGI) allows to evaluate improvements relating to other sexual domains different from the isolated DE, such as urinary disorders, chronic pelvic pain, and quality of life. With regard to sexual dysfunction, the questions contained in this questionnaire evaluate aspects such as libido and quality of ejaculation, which also had improved results.

Pharmacological treatment of patients with uncomplicated inflammatory MAGI is controversial. The literature has reported several pharmacological approaches with antibiotics (even with prostatic injections), alpha lithic, prostatic massage, and so on, but none of them found wide consensus in clinical practice [8]. The rationale for the use of phosphodiesterase V selective inhibitors in these patients could be attributed to possible relaxation of the prostatic smooth muscle and consequently, the increase of the prostatic tissue's ability to eliminate more rapidly the intraprostatic reflux products [8]. In clinical practice, the use of sildenafil (first molecule for this class) has been reported to determine the improvement of urinary symptom scores [9]. The enzyme NOS (nitric oxide synthase) is expressed in the transitional and peripheral regions of the prostate tissue, as shown by immunohistochemical studies [10]. In vitro, the localization of NOS within the prostatic smooth muscle has been reported [11]. Many years ago, it was suggested that the intraprostatic urinary reflux in the periurethral and transitional regions of the prostate represents a key dysfunctional element, responsible for the chronicity of the inflammatory process, which can also involve the peripheral zone of the gland [12]. Kirby et al. (the same first author 30 years after) reported the clinical case of a patient suffering from prostatodynia, controlled for 7 years with the use of morphine, intolerant to NSAIDs, and with complete resolution of symptoms after a period of treatment with Tadalafil 10 mg and 20 mg. After withdrawal of treatment, the patient reported reappearance of the same symptoms, so the patient was restarted treatment with Tadalafil 5 mg daily with new resolution of the symptoms. The authors report to have prescribed the same therapy in their clinical experience with the other patients with the same clinical features, with the same results [13]. In another clinical experience (a randomized, open-label, three-arm study), Liguori and colleagues evaluated the efficacy of a combined therapy with an alpha1-blockers (alfuzosin) and Tadalafil in 66 patients with lower urinary tract symptoms and ED. Results of this study showed that improvement in the maximum urinary flow rate was observed in all groups, but patients receiving combination therapy had greater improvement (29.6%) compared to patients treated only with alfuzosin (21.7%) or only Tadalafil (9.5%). Finally, the International Prostatic Symptom Score was significantly improved in patients treated with alfuzosin (27.2%) and was more marked with the combination therapy (41.6%), and a small increase, although not significant, was also observed on patients treated with Tadalafil (8.4%) [14].

Relative to the effects on spermatogenesis, the effectiveness of Tadalafil is very controversial. In the experimental model, it has been documented that rats exposed to increasing doses for 12 weeks (equivalent of 5, 10, and 20 mg daily) showed worsening of sperm parameters and a dose-dependent histopathological degenerative change of the seminiferous tubules and also in Johnsen's score (qualitative evaluation of spermatogenesis) was significantly lower in the animal treated with a dose of 1.8 mg/kg (equivalent to 20 mg) [15].

In a previous study, the acute administration of Tadalafil 20 mg, compared with the acute administration of sildenafil 50 mg, determined different effects on the percentage of spermatozoa with progressive motility. In particular, 1 and 2 hours after the administration of sildenafil, the authors observed a significant improvement in the progressive motility; on the contrary, after administration of Tadalafil, a significant reduction of the same parameter was observed [16]. The results of our study are not comparable for different reasons: different doses used (5 mg versus 20 mg), duration of the treatment (six months versus acute administration), and different clinical model (in our study, we enrolled patients with MAGI). In pathophysiological terms, the different modulations of sperm motility obtained 1 and 2 hours after administration suggest a pharmacological mechanism that could involve the secretion of prostate and seminal vesicles and not the epididymal phase of maturation [15]. Moreover, it should also be considered that Tadalafil in the therapeutic range plays also an inhibitory role on isoenzyme 11, involved in the modulation of spermatogenesis [17]. However, from a clinical point of view, the study of Hellstrom et al. showed that chronic daily use of Tadalafil 10 or 20 mg versus placebo was not associated with alterations of the sperm parameters [18]. Moreover, a recent Italian study conducted on 27 men aged between 19 and 35 years, suffering from psychogenic ED, and unaware of their fertility condition showed that after pharmacological treatment with Tadalafil 5 mg daily for 12 weeks, there was a significant improvement of sperm motility and their ejaculate volume [19]. Our results demonstrate that after treatment, only patients with PVE had a significant improvement in the progressive sperm motility. It is known that PVE represents the category with the lower quality of sperm parameters; moreover, it was demonstrated that the inflammatory dysfunction of the seminal vesicles contributes to further reduction of sperm motility, through different mechanisms, including the reduction of seminal concentrations of fructose [20]. In our previous study, we reported that after pharmacological treatment with Tadalafil, the ejection fraction of the seminal vesicles were significantly increased, suggesting a possible decontracting action of the treatment [21]. Moreover, the ultrasound evaluation allows to discriminate an “hypertrophic congestive” variant characterized by the dilation of the seminal vesicles (>14 mm) that represent the category with higher response to pharmacological treatment, other than the ultrasound variant called “fibro-sclerotic” associated with persistent low sperm quality after pharmacological treatment [22].

Another important aspect of the study concerns the possible improvements achieved by patients treated with Tadalafil regarding other sexual aspects, such as ejaculatory function and increased of libido. About the first aspect, in the literature, there is a growing interest and convergence for the possible use of Tadalafil in ejaculation disorders. In particular, the recent study by Ozcan et al. examined the intravaginal ejaculatory latency times (IELTs) of 30 men with lifelong premature ejaculation, treated for a month with Tadalafil 5 mg, showing a significant improvement in IELTs and plasma concentrations of nitric oxide after treatment [23]. Previously, Paduch et al. examined the ejaculatory and orgasmic function of patients with ED treated with Tadalafil. The authors evaluated 17 placebo-controlled trials lasting 12 weeks with the administration of Tadalafil at different doses (5, 10, and 20 mg), for a total of 2581 subjects. The study showed that Tadalafil 10 and 20 mg were associated with improvement of ejaculatory and orgasmic function in these patients [24]. Regarding the reduced percentage of patients who report painful ejaculation after therapy, there are no other clinical studies in the literature that examined the usefulness of treatment with Tadalafil. However, it is important to consider that the P is associated with painful ejaculation [25] and therefore, the overall improvement in symptoms could also lead this change. Actually, the main therapeutic option for the treatment of painful ejaculation is represented by alpha-lytic treatment [26]. Finally, the possible increase in serum testosterone levels' (aspect not evaluated in our study) consequence of increased sexual activity [27] or a direct and/or indirect action favored by the administration of Tadalafil [27] could justify the reduced percentage of patients with reduced libido and delayed ejaculation [28] after the treatment.

In our study, after treatment, through ultrasound evaluation, we observed an increase in the percentage of patients with uncomplicated MAGI and a reduction in the percentage of patients with complicated MAGI (P and PV). There are no other studies in the literature that have evaluated this specific aspect, which, however, assumes a particular importance, since it suggests the potential use of Tadalafil 5 mg daily for the secondary prevention of the chronicity of the inflammatory process and the possible complication of P in PV and/or PVE that are the categories with higher severity of signs and symptoms [4].

**Figure 1:**
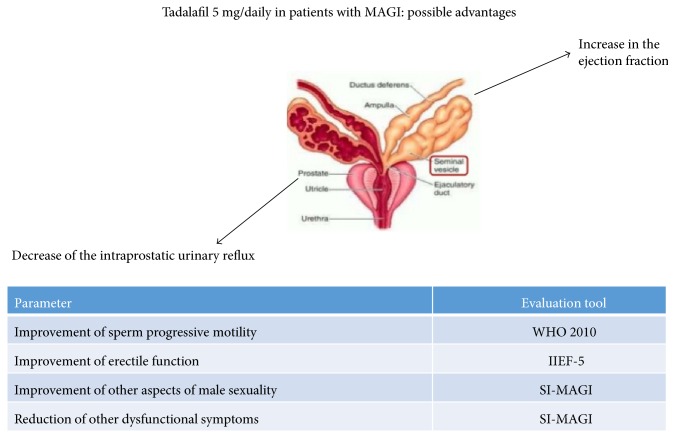
Tadalafil 5 mg/daily in patients with MAGI: possible advantages.

In conclusion, the results of this study suggest that therapy with Tadalafil 5 mg daily in patients with MAGI improves sexual function, sperm quality, and severity of symptoms and may prevent progression into complicated forms. The two main mechanisms that literature suggests to explain these effects could be the reduction of intraprostatic reflux [12] and the increased drainage of the seminal vesicles [21]. The absence of a control group is the main limitation of the study. Subsequent studies will have to confirm maintenance of the long-term effects.  Figure 1 summarizes the possible advantages of the treatment with Tadalafil 5 mg in patients with MAGI.

## Figures and Tables

**Table:** (a) Urinary disorders (levels of severity: absent: 0; mild: 1–6; moderate: 7–12; severe: 13–18)

	Question	0	1	2	3
1	Nocturia	0 times	1 time	2 times	>2 times
2	Urinary frequency	Absent	Every 2 hours	<2 hours	Every 1 hour
3	Strength of the urinary stream	Regular	Occasional difficulty	Constant difficulty	Constant difficulty and weakening voided urine
4	Quality of urinary stream	Regular	With intervals	Irregular and continuous	Post micturition drip
5	Failure to empty the bladder	Absent	Episodic	Frequent (>50%)	Constant
6	Duration of symptoms	Absent	<1 month	1–3 months	>3 months

**Table:** (b) Spontaneous and/or ejaculatory pain or discomfort (levels of severity: absent: 0; mild: 1–8; moderate: 9–16; severe: 17–24)

	Item	0	1	2	3
1	Perineal area	Absent	Occasional and/or <3 months	>50% or >3 months	Constant and by more than 3 months
2	Inguinal area	Absent	Occasional and/or <3 months	>50% or >3 months	Constant and by more than 3 months
3	Scrotal area	Absent	Occasional and/or <3 months	>50% or >3 months	Constant and by more than 3 months
4	Penile area	Absent	Occasional and/or <3 months	>50% or >3 months	Constant and by more than 3 months
5	Coccyx area	Absent	Occasional and/or <3 months	>50% or >3 months	Constant and by more than 3 months
6	Suprapubic area	Absent	Occasional and/or <3 months	>50% or >3 months	Constant and by more than 3 months
7	Irregular bowel and/or diarrhea and/or hematochezia	Absent	Presence of <1 symptom and/or sign	Presence of 2 symptoms/signs	Presence of 3 symptoms/signs
8	Duration of symptoms	Absent	<1 month	1–3 months	>3 months

**Table:** (c) Sexual function (levels of severity: absent: 0; mild: 1–11; moderate: 12–22; severe: 23–33)

	Item	0	1	2	3
1	Reduction of libido	Absent	Occasional and/or >3 months	>50% and/or >3 months	Constant and/or >3 months
2	Erectile dysfunction (achievement)	Absent	Occasional and/or >3 months	>50% and/or >3 months	Constant and/or >3 months
3	Erectile dysfunction (maintenance)	Absent	Occasional and/or >3 months	>50% and/or >3 months	Constant and/or >3 months
4	Difficulties in the second report	Absent	Occasional and/or >3 months	>50% and/or >3 months	Constant and/or >3 months
5	Premature ejaculation	Absent	Occasional and/or >3 months	>50% and/or >3 months	Constant and/or >3 months
6	Delayed ejaculation	Absent	Occasional and/or >3 months	>50% and/or >3 months	Constant and/or >3 months
7	Alteration of the macroscopic character of the ejaculate	Absent	One of the following: stringy, yellow or red-brown, volume reduction	Two of the following: stringy, yellow or red-brown, volume reduction	All of the following: stringy, yellow or red-brown, volume reduction
8	Painful ejaculation	Absent	Episodic	Frequent (>50%)	Constant
9	Ejaculate emission	Absent	Occasionally weakened	Weakened >50% of the time	Increasingly weakened
10	Prostatorrhea	Absent	Episodic	Frequent (>50%)	Constant
11	Hyperspermia	Absent	Episodic	Frequent (>50%)	Constant

**Table:** (d) Quality of life (levels of severity: absent: 0; mild: 1–5; moderate: 6–10; severe: 11–15)

	Item	0	1	2	3
1	Quality of life associated with the onset of symptoms	Like before	Satisfactions and failures	Frustrating and/or use of psychoanalysis	Terrible with recourse to psychoanalysis and/or drugs
2	Life changes associated with the onset of symptoms	Absent	Few	Several	Several and important
3	School or work days lost	0	1 day per month	2 days a month	> 2 days per month
4	Quality of married life since it began a diagnostic evaluation and/or treatment	Like before	Satisfactions and failures	Frustrating and/or use of psychoanalysis	Terrible with recourse to psychoanalysis and/or drugs
5	Infertility duration	<1 year	1-2 years	3-4 years	>4 years

**Table:** (e) Ultrasound criteria of MAGI

Prostatitis is suspected in the presence of >2 of the following ultrasonographic signs:
(1) asymmetry of the gland volume; (2) areas of low echogenicity; (3) areas of high echogenicity; (4) dilatation of periprostatic venous plexus; (5) single or multiple internal similar cystitis areas; and (6) area/s of moderate increased of vascularity (focal or multiple).

Vesiculitis is suspected in the presence of >2 of the following ultrasonographic signs:
(1) increase (>14 mm) anteroposterior diameter mono or bilateral; (2) asymmetry > 2.5 mm (normal 7–14 mm) compared to the contralateral vesicle; (3) reduced (<7 mm) anteroposterior diameter mono or bilateral; (4) glandular epithelium thickened and/or calcified; (5) polycyclic areas separated by hyperechoic septa in one or both vesicles; (6) fundus/body ratio > 2.5; (7) fundus/body ratio < 1; and (8) anteroposterior diameter unchanged after recent immediately ejaculation.

Epididymitis is suspected in the presence of >2 of the following ultrasonographic signs:
(1) increase in size of the head (craniocaudal diameter > 12 mm) and/or of the tail (craniocaudal diameter > 6 mm) (finding single or bilateral); (2) presence of multiple microcystis in the head and/or tail (finding single or bilateral); (3) low echogenicity or high echogenicity mono or bilateral; (4) large hydrocele mono or bilateral; (5) enlargement in the superior part of the cephalic tract and the superior/inferior part ratio > 1; and (6) unchanged anteroposterior diameter of tail after ejaculation.

**Table 2:** Diagnostic criteria of MAGI [1, 3–5, 19–21].

(a) History of urogenital infection and/or abnormal rectal palpation.
(b) Significant alterations in the expressed prostatic fluid and/or urinary sediment after prostatic massage.
(c) (1) Uniform growth of more than 10(3) pathogenic bacteria or more than 10(4) nonpathogenic bacteria per ml, in a culture of diluted seminal plasma. (c) (2) Presence of more than 10(6) (peroxidase positive) leucocytes per ml of ejaculate. (c) (3) Signs of disturbed secretory function of the prostate or seminal vesicles.
Diagnosis is accepted if at least 2 criteria are present:
(i) a + b, (ii) a + c [1or 2 or 3], (iii) b + c [1or 2 or 3], (iv) c1 + c2, (v) c1 + c3, (vi) c2 + c3.

**Table 3:** Change in symptom scores obtained after treatment with Tadalafil 5 mg daily for six months. *DOMAIN 1*: voiding disorders, *DOMAIN 2:* spontaneous and/or ejaculatory pain or discomfort; *DOMAIN 3:* sexual disorders; *DOMAIN 4:* quality of life.

	P	PV	PVE
DOMAIN 1 (T0)	10.0 ± 3.0	13.0 ± 2.0	17.0 ± 1.0^
DOMAIN 1 (T1)	5.0 ± 1.0^∗^	7.0 ± 2.0^∗^	7.0 ± 2.0^∗^

DOMAIN 2 (T0)	12.0 ± 3.0	18.0 ± 2.0	22.0 ± 2.0^
DOMAIN 2 (T1)	7.0 ± 2.0^∗^	10.0 ± 2.0^∗^	12.0 ± 2.0^∗^

DOMAIN 3 (T0)	10.0 ± 3.0	16.0 ± 2.0	26.0 ± 4.0^
DOMAIN 3 (T1)	4.0 ± 2.0^∗^	8.0 ± 2.0^∗^	11.0 ± 4.0^∗^

DOMAIN 4 (T0)	7.0 ± 3.0	10.0 ± 2.0	14.0 ± 1.0^
DOMAIN 4 (T1)	3.0 ± 2.0^∗^	5.0 ± 2.0^∗^	6.0 ± 2.0^∗^

^∗^ *p* < 0.001 versus T0; ^*p* < 0.05 versus P and PV.

**Table 4:** Main sperm parameters examined before and after pharmacological treatment with Tadalafil 5 mg daily for six months.

	P	PV	PVE
Volume (T0) ml	2.2 ± 1.1	1.9 ± 1.1	1.8 ± 1.0
Volume (T1) ml	2.4 ± 1.2	2.1 ± 1.2	2.0 ± 1.2

Density (T0) mil/ml	33.0 ± 8.0	26.0 ± 8.0	18.0 ± 8.0
Density (T1) mil/ml	36.0 ± 6.0	29.0 ± 9.0	24.0 ± 7.0

Normal forms (T0) %	10.0 ± 4.0	9.0 ± 3.0	9.0 ± 4.0
Normal forms (T1) %	12.0 ± 6.0	10.0 ± 4.0	10.0 ± 3.0

Total motility (T0) %	32.0 ± 9.0	30.0 ± 8.0	16.0 ± 8.0
Total motility (T1) %	36.0 ± 7.0	33.0 ± 6.0	30.0 ± 6.0^∗^

Prog. motility (T0) %	25.0 ± 5.0	24.0 ± 8.0	10.0 ± 8.0
Prog. motility (T1) %	28.0 ± 4.0	26.0 ± 6.0	25.0 ± 6.0^∗^

Leukocytes (T0) mil/ml	1.2 ± 0.6	1.4 ± 0.4	1.5 ± 0.6
Leukocytes (T1) mil/ml	1.0 ± 0.3	1.1 ± 0.3	1.2 ± 0.4

^∗^ *p* < 0.001 versus T0.

**Table 5:** Redistribution of categories of MAGI (P: prostatitis; PV: prostate-vesiculitis; PVE: prostate-vesciculo-epididymitis) evaluated according to ultrasound criteria before and after treatment with Tadalafil 5 mg daily for six months.

	P	PV	PVE
Baseline	68	30	22
After treatment	90^∗^	20^∗^	10^∗^

^∗^ *p* < 0.001 versus T0.

**Table 6:** Percentage of the improvement reported to question numbers 1, 4, 5, 6, and 8 of the domain regarding sexual function assessed through the SI-MAGI questionnaire.

	Item	Baseline	After treatment
1	Reduction of libido	30/120 (25.00%)	12/120 (10.00%)^∗^
4	Difficulties in the second report	60/120 (50.00%)	30/120 (25.00%)^∗^
5	Premature ejaculation	10/120 (8.00%)	5/120 (4.00%)^∗^
6	Delayed ejaculation	15/120 (12.50%)	10/120 (8.00%)^∗^
8	Painful ejaculation	12/120 (10.00%)	5/120 (4.00%)^∗^

^∗^ *p* < 0.001 versus T0.
